# Airway Management of Orofacial Infections Originating in the Mandible

**DOI:** 10.3390/jpm13060950

**Published:** 2023-06-04

**Authors:** Andreas Sakkas, Christel Weiß, Wolfgang Zink, Camila Alejandra Rodriguez, Mario Scheurer, Sebastian Pietzka, Frank Wilde, Oliver Christian Thiele, Robert Andreas Mischkowski, Marcel Ebeling

**Affiliations:** 1 Department of Cranio-Maxillo-Facial-Surgery, University Hospital Ulm, 89081 Ulm, Germany; 2 Department of Cranio-Maxillo-Facial-Surgery, German Armed Forces Hospital Ulm, 89081 Ulm, Germany; 3 Medical Statistics and Biomathematics, Mannheim Medical Faculty of the Heidelberg University, 68167 Mannheim, Germany; 4 Department of Anesthesiology and Intensive Care Medicine, Ludwigshafen Hospital, 67063 Ludwigshafen, Germany; 5 Department of Anesthesiology and Intensive Care Medicine, University Hospital Ulm, 89081 Ulm, Germany; 6 Department of Cranio-Maxillo-Facial-Surgery, Ludwigshafen Hospital, 67063 Ludwigshafen, Germany

**Keywords:** airway management, tracheostomy, intubation, orofacial infection, odontogenic abscess, mandibular infections

## Abstract

The primary aim of this study was to assess the incidence of a difficult airway and emergency tracheostomy in patients with orofacial infections originating in the mandible, and a secondary aim was to determine the potential predictors of difficult intubation. This retrospective single-center study included all patients who were referred between 2015 and 2022 with an orofacial infection originating in the mandible and who were surgically drained under intubation anesthesia. The incidence of a difficult airway regarding ventilation, laryngoscopy, and intubation was analyzed descriptively. Associations between potential influencing factors and difficult intubation were examined via multivariable analysis. A total of 361 patients (mean age: 47.7 years) were included in the analysis. A difficult airway was present in 121/361 (33.5%) patients. Difficult intubation was most common in patients with infections of the massetericomandibular space (42.6%), followed by infections of the mouth floor (40%) and pterygomandibular space (23.5%). Dyspnea and stridor were not associated with the localization of infection (*p* = 0.6486/*p* = 0.4418). Multivariable analysis revealed increased age, restricted mouth opening, higher Mallampati scores, and higher Cormack–Lehane classification grades as significant predictors of difficult intubation. Higher BMI, dysphagia, dyspnea, stridor and a non-palpable mandibular rim did not influence the airway management. Patients with a difficult airway were more likely to be admitted to the ICU after surgery than patients with regular airway were (*p* = 0.0001). To conclude, the incidence of a difficult airway was high in patients with orofacial infections originating in the mandible. Older age, limited mouth opening, a higher Mallampati score, and a higher Cormack–Lehane grade were reliable predictors of difficult intubation.

## 1. Introduction

Odontogenic infections are caused by untreated dental caries, periodontal disease, or trauma and are a common reason for seeking medical care in oral and maxillofacial surgery units [1,2,3]. Data from developed countries suggest that the rate and severity of odontogenic infections in patients who present to hospital emergency departments are increasing [4,5]. Immediate surgical incision and drainage combined with intravenous administration of antibiotics remains the standard treatment [6,7].

If the primary cause of orofacial infections in the mandible spaces is not promptly eliminated, swelling into the cervical multilayered deep fascia can occur, leading to infection in the deep neck space [8]. The severity of these infections can be decreased via surgical clearance and antibiotics, but if the infection spreads, it can obstruct the airway, leading to morbidity and, in rare cases, mortality [8,9,10,11]. Extended orofacial infections are challenging because of the complex anatomy of the head and neck, the vicinity of vital structures, and the risk of the infection spreading to adjacent spaces. The location of the infection can also affect airway management, and concomitant trismus can hinder conventional laryngoscopy and intubation [11,12]. The risk of airway compromise is particularly high in patients with infections in several musculofascial spaces [6].

Airway management in patients with abscesses in the perimandibular space is challenging because of swelling, restricted mouth opening, and neck stiffness. Further risk factors for anesthesia-related complications include stridor, neck operations, dysgnathia, restricted head reclination, a reduced thyreomental distance, sleep apnea syndrome, and Mallampati grade III or IV, and these factors have to be considered carefully [13]. Appropriate preoperative assessment and a safe airway are the hallmarks for successful treatment and fewer morbidity-related complications [14]. Modern airway management, including video laryngoscope and fiberoptic methods, can achieve successful intubation even in challenging cases. However, the specific demographic and clinical features that indicate a difficult airway need to be recognized early on. Accurate prediction of a difficult airway has also been associated with successful intubation on the first attempt [15].

Faster and safer surgical treatment reduces the inpatient hospital stay and thereby the economic burden on the healthcare system. Thus, a multidisciplinary approach involving oral and maxillofacial surgeons, anesthesiologists, and emergency medicine physicians is essential for a successful outcome. Although the clinical presentation and treatment outcomes of odontogenic infections have been well reported, the airway management of patients with extended infections originating in the mandible has not been well studied [1,2,4,16]. Since exploration and advancement in personalized medicine is a rapidly growing domain nowadays, distinguishing the airway management of an individual with mandible-related orofacial infection from others with similar clinical presentations can improve diagnosis, reduce complications, and develop outcomes [17].

The primary aim of this study was to assess the incidence of a difficult airway and emergency tracheostomy in patients with orofacial infections originating in the mandible, and a secondary aim was to determine potential predictors of difficult intubation. The length of hospitalization, incidence of surgical revisions, and rate of in-hospital mortality were also evaluated retrospectively.

## 2. Materials and Methods

For this observational retrospective single-center study, we reviewed the medical records of all patients with orofacial infections/abscesses originating in the mandible who were surgically treated under general anesthesia in our department of oral and plastic maxillofacial surgery between January 2015 and August 2022. Records were retrieved from our hospital electronic database. Ethical approval for this study was obtained from the ethics committee of the chamber of physicians in Rhineland-Palatine, Mainz, Germany (approval number: 2022-16439, approval date: 8 April 2022), and the study was performed in accordance with the Declaration of Helsinki 1964 and its later amendments (World Medical Association, Declaration of Helsinki).

We enrolled patients with an orofacial infection/abscess in the perimandibular spaces that originated in the mandible and who underwent surgical drainage through a cervical approach under intubation anesthesia. Patients were excluded if the orofacial infection did not involve the mandible or adjacent areas, if they underwent surgical drainage under local anesthesia, if no surgical intervention was performed, or if their medical charts were incomplete.

### 2.1. Patient Screening

Our standard clinical protocol included clinical assessment, laboratory tests, and an initial computer tomography (CT) scan. Patients with swelling around the mandibular area were clinically evaluated for dysphagia, dyspnea, stridor, the palpability of the caudal mandibular rim, and mouth opening. Any clinical information not in the emergency department report was assumed as negative. Extraoral surgical drainage under general anesthesia was indicated by a board-certified oral and maxillofacial surgeon after the evaluation of the clinical and radiological findings. No cases of intraoral drainage were documented.

Extraoral surgical drainage was performed with a submandibular incision in the cervical skin fold, two finger widths below the mandibular rim to protect the marginal branch of the facial nerve, in the direction of the nerve. After blunt preparation of the mandibular rim using blunt dissecting scissors, the lingual and vestibular/submental margins were exposed under constant bone contact. After pus was discharged, a microbiological smear was taken and the wound cavity was irrigated with a disinfecting agent. Two drainage tubes were inserted with grain forceps and fixed with skin sutures. The cause of the infection was also eliminated. All patients received intravenously administered antibiotics from the time of admission until home discharge.

The removal of the intraoral source of infection was addressed in the same or secondary operation, depending on the specific cause and its extension. In cases of odontogenic focus caused by chronic apical parodontitis, the infected teeth were extracted always simultaneously to the extraoral surgical drainage. Tooth extractions were performed only under intubation anesthesia. When the infective cause was an ARONJ or osteomyelitis, sequestrectomy and bone decortication were performed secondarily in the same or a further in-patient hospital stay. Orofacial abscesses following osteotomies, dental implantations or osteosynthesis were treated only with extraoral surgical drainage, mostly under intubation anesthesia but also with a laryngeal mask when possible.

A difficult airway was defined by the presence of difficult ventilation, difficult laryngoscopy, or difficult intubation, according to the current German guidelines [13]. A difficult airway was defined after traditional mask ventilation and intubation using direct laryngoscopy was attempted by a board-certificated anesthetist. Ventilation using the face mask or an extraglottic airway device was defined as difficult or impossible if ventilation was insufficient or completely unsuccessful because of leakage or/and resistance during inspiration or expiration. Ventilation was also defined as difficult if several attempts were needed to place the extraglottic airway device. Difficult laryngoscopy was defined as Cormack–Lehane grade of III or IV and was identified via direct laryngoscopy [18]. The laryngoscopic view was assessed and graded according to the final intubation attempt after each intubation was finished. Difficult endotracheal intubation was defined by the need for several intubation attempts.

Emergency tracheotomy was performed in an awake setting in patients with a clearly compromised airway, who could either be sufficiently ventilated or intubated preoperatively. A secondary tracheotomy was performed during the post-operative stay at the intensive care unit (ICU) electively in patients with resistant soft tissue swelling and high infection parameters, who required prolonged intubation. All tracheotomized patients were decanulated according to regressed soft tissue swelling and normal respiratory condition, and the tracheostoma was primarily surgically closed before discharge. Primary tracheotomized patients and patients with a difficult airway, concomitant comorbidities and extended orofacial infection who required advanced respiratory support were admitted at the ICU post-operatively for further respiratory monitoring and surveillance. Patients who were admitted at the surgical ward post-operatively were closely monitored by the nursing staff via continuous control of the oxygen saturation and respiratory condition including supporting oxygen administration if needed. The attended oral and maxillofacial surgeon also re-evaluated the patient closely to ensure a safe post-operative course. In case of a respiratory emergency (e.g., desaturation, shallow respiration and shortness of breath), the anesthesiologic team was promptly informed.

### 2.2. Data Collection

Data were collected from patients’ electronic hospital charts and patients were anonymized before data analysis. Extracted data comprised patient age, patient gender, antithrombotic medication, body mass index (BMI), the localization of the infection, clinical symptoms at admission (dysphagia, dyspnea, and stridor), relevant clinical findings (palpability of the mandibular rim and extension of mouth opening), etiology of the abscess, ASA grade, Mallampati score, Cormack–Lehane grade, airway management (difficult/regular), the ventilation method, laryngoscopy and intubation (difficult/regular), and the outcome (admission to the ICU, length of hospitalization, rate of surgical revision, or in-hospital mortality) [18,19]. The Mallampati score or Cormack–Lehane grade could not be ascertained if direct intraoral visualization was not possible because of a restricted mouth opening.

We collected all CT scans prescribed by the attending clinician after the clinical evaluation. To measure interrater reliability, each case was interpreted by two board-certified radiologists. We abstracted all radiological findings that were relevant to abscess formation in the spaces around the mandible (paramandibular, submandibular, perimandibular, mouth floor, submental, massetericomandibular, pterygomandibular, and parapharyngeal space). The exact diagnosis and localization of the infection were determined from the operation and radiological report.

### 2.3. Statistical Analysis

Data were centralized in an electronic format using Microsoft Excel software and analyzed descriptively. Statistical analysis was performed using SAS^®^, Release 9.4 software (SAS Institute Inc., Cary, NC, USA). Descriptive statistics were used to describe baseline patient characteristics. All categorical variables were expressed as absolute values (*n*) and relative incidences (%). For metric variables, the standard deviation was calculated. A multivariable analysis was performed to find associations between the possible influencing variables and a difficult airway. Associations between categorical variables were described with cross-tabulations, and chi-square tests were used to investigate a potential association between infection localization, clinical features and a difficult airway. Fisher’s exact test was used to compare smaller subgroups. The Cochran–Armitage trend test was used to detect associations between age, BMI, Mallampati score, Cormack–Lehane grade, and the intubation modality. A t-test was used to compare the length of hospital stay in tracheotomized and non-tracheotomized patients. A two-sided *p* value of less than 0.05 was considered statistically significant.

## 3. Results

### 3.1. Demographic Distribution

A total of 361 patients were included in the analysis. There were more males (196/361; 54.3%) than females (165/361; 45.7%) and the male:female ratio was 1.18:1. The patients’ age at the time of injury was 5–92 years, and the mean ± SD age was 47.75 ± 19.57 years. Most patients (46.8%) were older than 50 years. The mean ± SD BMI at the time of admission was 26.8 ± 6.59. The baseline patient characteristics are presented in Table 1.

### 3.2. Etiology and Localization of Infection

The most common etiology of infection was chronic apical periodontitis (*n* = 270; 74.8%), followed by infections post-osteotomy (*n* = 60; 16.6%). Infections were most common in the perimandibular space (*n* = 168; 46.5%), followed by the submandibular space (*n* = 111; 30.7%) and the submental space (*n* = 36; 10%) (Table 1). Dysphagia was detected significantly more often in patients with submental infections (Fischer’s exact test: *p* = 0.0065), while a non-palpable mandibular rim was documented significantly more often in patients with infections of the submandibular space (Chi-square test: *p* < 0.0001), perimandibular space (Chi-square test: *p* < 0.0001), mouth floor (Fischer’s exact test: *p* = 0.0002), submental area (Chi-square test: *p* = 0.0373), and parapharyngeal area (Fischer’s exact test: *p* < 0.0001). Dyspnea and stridor were not associated with the localization of infection (Fischer’s exact test: *p* = 0.6486 and *p* = 0.4418, respectively). 

### 3.3. Airway Management

Cervical surgical drainage was performed within the first 24 h after admission in all patients. 

**Figure 1:**
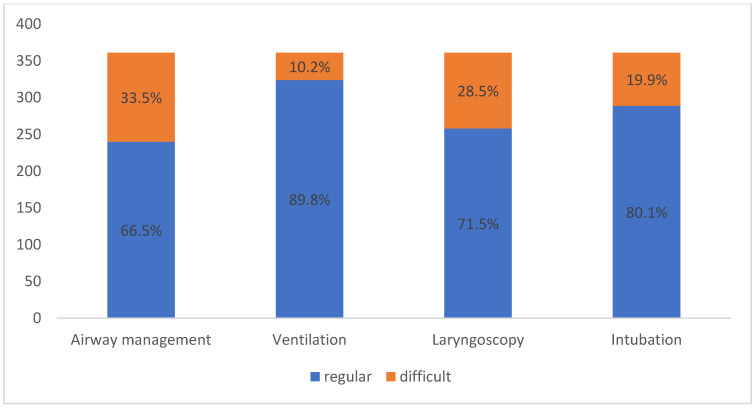
Airway modality regarding ventilation, laryngoscopy and intubation according to the study’s definition criteria of a difficult airway.

Ventilation was defined as difficult in 10.2% (*n* = 37/361) of patients and an extraglottic airway device was used. Laryngoscopy was difficult in 28.5% (*n* = 103/361) of patients and intubation was difficult in 19.9% (*n* = 72/361) of patients. A difficult airway was documented in 121 (33.5%) patients (Figure 1).

**Figure 2:**
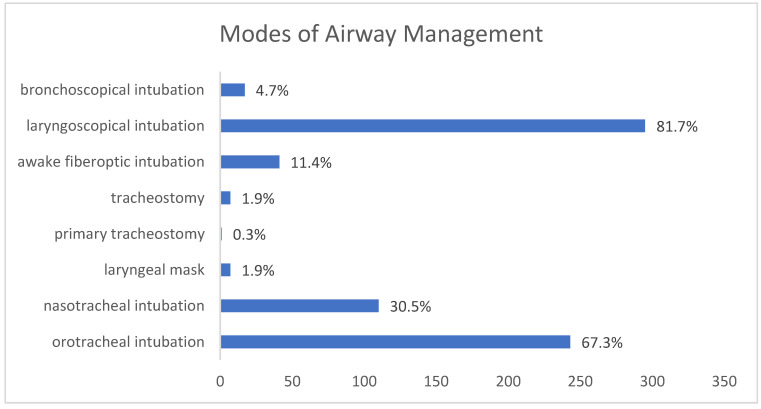
Different modes of airway management in the study population.

One hundred and ten patients (30.5%) underwent nasotracheal intubation, 243 patients (67.3%) underwent orotracheal intubation and seven patients (1.9%) were fitted with a laryngeal mask. One patient (0.3%) with a paraphyryngeal abscess was primarily tracheotomized in an awake setting by a “cannot intubate, cannot ventilate” situation. In patients anesthetized with a laryngeal mask, only extraoral surgical drainage was performed without intraoral intervention. Rapid-sequence induction was performed in seven patients (1.9%) because of extensive swelling and progressive dyspnea. Intubation was laryngoscopic in 295 patients (81.7%), bronchoscopic in 17 patients (4.7%), and awake-fiberoptic in 41 patients (11.4%) (Figure 2).

Of the patients, 345 (95.5%) were extubated immediately after surgery and 16 patients (4.5%) remained intubated and were admitted to the ICU for further observation. Secondary tracheotomy was performed in six (1.6%) patients during the ICU stay because of resistant swelling and the need for prolonged intubation.

Of 121 patients with a difficult airway, 10.5% (*n* = 13) required post-operative monitoring in the ICU and 89.5% (*n* = 108) were transferred to the normal surgical ward. Only 1.2% of patients with regular airway management (*n* = 3/240) needed intensive care, and 98.8% (*n* = 237/240) were transferred to the normal surgical ward (chi-square test: *p* = 0.0001).

### 3.4. Multivariable Analysis

The multivariable analysis revealed older age, restricted mouth opening, higher Mallampati scores, and higher Cormack–Lehane classification grades as significant predictors of difficult intubation.

Difficult intubation was documented in 5/79 (6.3%) patients younger than 30 years of age, in 22/113 (19.4%) patients aged between 30 and 50 years, and in 45/169 (26.6%) patients older than 50 years. Increasing age significantly increased the risk of difficult intubation (Cochran–Armitage trend test: *p* = 0.0002). Difficult intubation was detected in 17.5% (28/160) of patients with a BMI of < 24.9, in 19.8% (21/106) of patients with a BMI of 25–29.9, and in 24.2% (23/95) of patients with a BMI of ≥ 30 (Cochran–Armitage trend test: *p* = 0.2013).

Difficult intubation was most common in patients with infections of the massetericomandibular space (42.6%), followed by patients with infections of the mouth floor (40%) and pterygomandibular space (23.5%), but these differences were not significant (*p* > 0.05) (Table 2).

We also examined the correlation between patient’s symptoms at the initial examination and the occurrence of difficult intubation (Table 3). Of the patients, 25% (*n* = 3/12) with dysphagia and 25% (*n* = 3/12) with dyspnea experienced a difficult intubation. A difficult intubation was also observed in two out of four (50%) patients with initial stridor. Difficult intubation occurred in 21% (62/295) of the patients in whom the mandibular rim could not be consistently palpated during clinical examination and in 15.2% (*n* = 10/66) of patients with a consistently palpable mandibular rim. Difficult intubation was detected in 18.9% of patients with a restricted mouth opening (*n* = 66/348). Only a restricted mouth opening was significantly associated with an increased rate of difficult intubation.

Higher Mallampati scores and Cormack–Lehane classifications were significantly associated with an increased risk of difficult intubation (Cochran–Armitage trend test: *p* < 0.0001) (Table 4).

### 3.5. Post-operative Outcome

**Figure 3:**
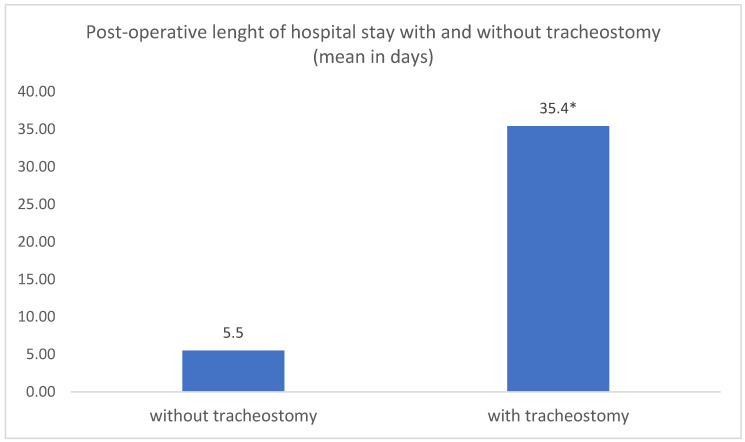
Comparative analysis of mean post-operative length of hospital stay in patients with and without tracheostomy. * *t*-test: *p* = 0.0194.

The mean post-operative length of hospitalization was 6.08 days (range: 1–66 days). The length of stay was significantly shorter for patients without a tracheostomy (mean: 5.5 days; range: 1–20 days) than for tracheotomized patients (mean: 35.4 days; range: 9–66 days) (*t*-test: *p* = 0.0194) (Figure 3).

Of the 121 patients with a difficult airway, 10.5% required monitoring in the ICU and 89.5% were transferred to the normal surgical ward after surgery. Only 1.2% of patients with regular airway management required intensive care; the remaining 98.8% (*n* = 237/240) were transferred to the normal surgical ward (Fischer’s exact test: *p* < 0.0001). In total, 11 out of 72 patients (15.3%) with a difficult intubation and 5 out of 189 patients (1.7%) with a regular intubation were admitted post-operatively to the ICU for further observation (Fischer’s exact test: *p* < 0.0001). The 16 intubated patients were treated in the ICU for a mean period of 6.6 days (range: 1–23 days). Seven of the tracheotomized patients stayed for a mean period of 11.7 days (range: 1–23 days) and nine of the patients without tracheostomy stayed for a mean period of 2.7 days (range: 1–5 days).

The in-hospital mortality rate was 0.3% (*n* = 1/361). A 56-year-old male with a parapharyngeal infection of unknown origin who initially presented with dysphagia, dyspnea, and stridor died 3 days post-operatively after developing cardiogenic shock. Twenty-two patients (6.1%) underwent surgical re-drainage during hospitalization because of persistent swelling and elevated laboratory infection parameters.

## 4. Discussion

We aimed to specify the clinical features that predict a difficult airway and difficult intubation in patients with orofacial infections originating in the mandible. Our results provide valuable insights into how preoperative evaluation of these patients can increase the safety of airway management during surgical treatment of orofacial infections. Considering the challenges and features of personalized medicine and dentistry nowadays, through this article we aimed to apply our collective knowledge with regard to precise risk factors to enable an individualized therapy and develop precision health care [17].

Most reported orofacial infections have an odontogenic cause, and chronic apical periodontitis was the most common cause of infection in our study [1,2,3,4]. This is in agreement with the findings of Tapiovaara and Kinzer et al., who found that odontogenic infections can lead to necrotizing fasciitis with a mediastinal extension [11,20]. Postsurgical infection (such as after osteotomies, dental implantations, or trauma surgery) was the next most common cause of infection in our cohort, followed by antiresorptiva-related osteonecrosis of the jaw. These findings show a high possibility of severe infection after oral surgery; therefore, we recommend intensive post-operative care and evaluation to avoid this, especially in immunosuppressed patients.

The affected anatomic space is important for the surgical and anesthesiologic treatment of an infection. In our study, more than 75% of infections were detected in the perimandibular and submandibular space. These spaces are likely commonly affected by infection because of their close anatomic relationship with the roots of the posterior mandibular molars [1]. However, contrary to our initial hypothesis, the localization of infection was not correlated with an increased risk of difficult intubation. We agree with Bowe et al., that trismus is more severe when more spaces are involved [1]. Infection of the masticatory muscles can reduce mouth opening, thereby restricting intraoral access for anesthesiologic management. In these cases, fiberoptic intubation and an experienced consultant anesthetist is needed. In our study, 11.4% of the patients needed awake fiberoptic intubation. This was decided for patients with extended abscesses and combined trismus, who had a history of a difficult airway after previous operations or with a clear risk of an impossible direct laryngoscopy determined in advance. We encourage awake fiberoptic intubation in high-risk patients because intubation can be performed prior to the induction of general anesthesia with its possible risks of inadequate oxygenation, loss of upper airway consistency, and failed intubation. However, an adequate time for preparation and a cooperative patient are required for a safe and successful procedure. We agree with Bowe et al., that using CT scans to monitor orofacial infections is a valuable way to indicate potential airway compromise [1]. Based on these observations, we recommend close preoperative communication between anesthesiologists and surgeons to guarantee the best post-operative outcome, especially in cases of perimandibular and submandibular abscesses.

Surgical drainage of abscesses in the lower orofacial spaces is a short procedure, and the airway tract is often edematous, which increases the risk of airway compromise [14]. Careful preoperative assessment is crucial in these patients to select the appropriate airway modality. Clinical features for assessing airway difficulty include the mouth opening, the Mallampati score, palpability of the mandibular rim, and symptoms such as dysphagia, dyspnea, and stridor. All patients in this study were referred with definitive infection-related swelling that required surgical drainage. Cervical access was necessary for drainage because the extended trismus prevented intraoral access.

In our study, most intubations were laryngoscopic and 33.5% of patients had a compromised airway. These findings cannot be compared with those of other studies because the published definitions of a difficult airway vary widely [13]. Problems during endotracheal intubation are often referred to as “difficult intubation” without distinguishing between “laryngoscopy” and “intubation”. These terms need to be separated because anatomical and optical axes converge in laryngoscopy and an acceptable laryngoscopic result can lead to a successful intubation. The rate of difficult ventilation was 10.2% in our study, which is higher than the rates published in other studies [13,21,22]. We also reported a higher rate of difficult direct laryngoscopy (28.5%) than did other studies (1.5–13.45%) [13,23,24]. However, our findings cannot be directly compared with those of other studies because of differences in protocol and patient sample.

We performed surgery under laryngeal mask airway in only seven cases. These patients received extraoral drainage of abscesses following dental implantations or dentoalveolar procedures without further intraoral intervention. The use of a laryngeal mask can be useful in emergency cases of difficult or impossible intubation, when the patient appears to be in stable condition, and the duration of the planned surgical procedure is quite short. However, this airway management method does not support intraoral interventions and should not be preferred when the intraoral infective source has to be removed simultaneously.

We had one unexpected “cannot intubate, cannot ventilate” situation in our cohort, in a patient with a paraphyryngeal abscess [13,23]. This patient received a primary tracheostomy. This is similar to the findings of Motahari et al. and Kataria et al., who reported peritonsillar abscesses requiring primary tracheostomy in 1% and 5% of patients [25,26]. We did not observe enough tracheostomy cases to determine the correlation between the localization of infection and rate of tracheostomy. In our cohort, 6/16 patients who were admitted to the ICU were tracheotomized because of persistent swelling and prolonged intubation. We agree with Tapiovaara et al., that tracheostomy can avoid laryngeal injury in patients needing prolonged intubation, thereby reducing the need for sedation and mechanical ventilation [11]. Early tracheostomy has been correlated with lower mortality rates in patients with deep neck infections [27]. However, we believe that tracheostomy can be avoided by proper preoperative airway planning. If emergency intubation is needed, for example in more challenging patients, we recommend securing the patient’s airway via awake intubation and video laryngoscopy under sedation by experienced anesthesiologists.

We identified four predictors of difficult intubation: older age, limited mouth opening, a higher Mallampati score, and a higher Cormack–Lehane grade. These predictors may be able to be used to identify patients with difficult intubation following either conventional direct laryngoscopy or indirect laryngoscopy with fiberoptic methods. These factors can also be used to identify patients at risk of a difficult airway in the preoperative anesthesiologic assessment. Unexpectedly, a higher BMI was not associated with an increased rate of difficult intubation. Similarly, dysphagia, dyspnea, stridor and a non-palpable mandibular rim as clinical symptoms of a difficult airway that could be assumed in advance were also not correlated to higher risk for difficult intubation. Further studies are needed to develop an intubation prediction score for determining the risk of difficult intubation in patients with orofacial infections.

We found that difficult intubation was more common in patients older than 50 years than in younger patients. This finding is consistent with that of previous studies that have shown an association between increased age and difficult airway management [21,22,24]. The increased risk of a difficult airway in older individuals could be due to age-related changes in the airway anatomy of these individuals, such as decreased elasticity, decreased muscle tone, and decreased neck mobility. The increased risk of difficult intubation in older individuals may be due to the anterior shift of the mandible relative to the maxilla caused by the attrition of the molar cusps and the regeneration of the cementum [28]. Intubation difficulties may also be caused by age-related disc displacement and osteoarthritis of the temporomandibular joint, which can restrict mouth opening, as well as by mandibular resorption and alveolar remodeling, which can cause jaw retraction and drooping, making mask ventilation and laryngoscopy more difficult [28]. Kyphotic deformities and poor neck mobility may also contribute to this problem [21,22,24]. In addition, fibroblast proliferation decreased with age, which reduces the flexibility and elasticity of the oral cavity, thereby limiting mouth opening. This may also contribute to difficult airway management in older individuals [29].

The risk of difficult intubation was higher in patients with a BMI of ≥ 30. These findings are consistent with those of previous studies [30,31]. Difficult airway management in obese patients may be caused by the larger neck circumference, increased fat deposition in the upper airway, and decreased lung volume in these individuals. We also observed a significantly higher rate of difficult intubation in underweight patients in out cohort, likely due to the smaller dimensions of the lower face.

Surprisingly, we found no correlation between the localization of the infection and modality of the intubation. We expected patients with abscesses in the mouth floor, pterygomandibular space, or parapharyngeal space to have a more demanding airway because of tissue edema, an obstructed upper airway, and jaw immobility, but this was not confirmed—possibly because we only had a few patients with these infections in our cohort. As expected, patients with paramandibular and submental abscesses had the lowest risk of having a difficult airway, likely because these spaces are anatomically more distant from the respiratory tract and mouth-opening muscles.

The incidence of difficult intubation was higher in patients with higher Mallampati scores and Cormack–Lehane grades, which is in accordance with published findings [13]. We did not find any association between clinical symptoms (such as dysphagia, dyspnea, and stridor) and intubation modality, possibly because of the small number of patients with these symptoms. Only limited mouth opening was associated with increased intubation difficulty, which has been confirmed in past studies [13,24]. However, we recommend examining patients carefully for these symptoms as they may predict difficult or impossible direct laryngoscopy or intubation.

As expected, difficult airway management was a significant risk factor for ICU admission after surgery. In our cohort, the rate of post-operative admission to the ICU was 4.4% and the mean ICU stay length was 6.6 days. This was higher than the ICU admission rate of 0.4% and longer than the mean ICU stay length of 4 days reported by Bowe [1]. Tapiovaara et al. reported a longer median ICU stay of 7 days [11]. Case numbers were too low to detect a correlation between the localization of infection and the duration of ICU stay.

Patients who underwent tracheostomy had a significantly longer hospital stay than did patients who did not undergo tracheostomy. This can be explained by the significantly advanced disease that necessitates tracheostomy in the first place [11,32,33]. In support of our findings, Nagarkar et al. also found a significantly longer hospital stay following tracheostomy, but this was following head and neck cancer surgeries [14].

Delaying surgical treatment of orofacial infections can make anesthesiologic treatment more challenging and increase the overall duration of hospital stay. Coexisting medical conditions can also increase the risk of severe infection or sepsis. Most studies recommend the immediate surgical drainage of abscesses combined with the intravenous administration of antibiotics in odontogenic or deep neck infections; however, the optimal timing of surgery is still under debate [2,7,34,35]. All patients in this cohort were operated on within the first day of admission. In concordance with others, we strongly recommend early surgical drainage and the simultaneous intravenous administration of antibiotics to avoid compromising the airway further [36,37].

There are some limitations to this study. First, the study was restricted to one emergency care unit so the results may not be generalizable to other centers. Second, the retrospective nature of the research may have caused documentation bias; however, this limitation was outweighed by the large study cohort. Third, the different level of education and experience of the treating physicians in our study may have biased our results. Data were collected from medical records completed by the intubator, which could have caused observer bias with regard to the intubation approach. As a result, the choice of airway management and its difficulty could only be evaluated subjectively. Fourth, several evidence-based clinical predictors of a difficult airway, such as previous operations, radiation of the head/neck area, sleep apnea syndrome, mandibular protrusion, thyreomental distance, and macroglossia could not be extracted from the patients’ files. Fifth, we did not compare our patient sample with a control cohort of patients with orofacial infections originating in the maxilla or other anatomic spaces, so our findings are limited to infections in the mandible. Sixth, we included patients of all ages, so our results cannot be generalized to a specific age group. Finally, we did not evaluate the influence of the microbiological examination of pus swabs and of systemic health on the duration of hospitalization in our study. This could have introduced a bias to our findings since the length of stay does not depend only on airway management [38]. Future prospective studies with standardized protocols are warranted to validate our preliminary findings.

## 5. Conclusions

The incidence of a difficult airway was 33.5% in our cohort of patients with orofacial infections originating in the mandible. Factors increasing the risk of a difficult intubation were older age, limited mouth opening, higher Mallampati scores, and higher Cormack–Lehane grades. We recommend the careful evaluation of these factors preoperatively as a way of reliably predicting difficult intubation. Difficult intubation was most common in patients with infections of the massetericomandibular space, followed by infections of the mouth floor and pterygomandibular space, albeit without statistical significance. A higher BMI, dysphagia, dyspnea, stridor and a non-palpable mandibular rim did not influence airway management. We highlight the importance of clear communication between surgeons and anesthesiologists to determine the safest airway approach during surgery. Fiberoptic orotracheal intubation is the most appropriate technique for managing a difficult airway in patients with these orofacial infections. Although tracheostomy is both rare and safe, individual assessment and proper preoperative planning is required. The modalities of airway management and tracheostomy have a significant impact on the post-operative length of hospital stay. These findings will help clinicians to reduce the risk of complications and improve patient safety during airway management. Our preliminary results and recommendations should be confirmed by well-designed prospective studies.

## Figures and Tables

**Table 1:** Baseline demographics, clinical and anesthesiologic findings and outcome of the overall study population.

	Study Population	
	*n*	%
Total	361	100.0%
Gender		
male	196	54.3%
female	165	45.7%
Age		
<30 years	79	21.9%
≤30–50 years	113	31.3%
≥50 years	169	46.8%
BMI		
<24.9	160	44.3%
≤25–29.9	106	29.4%
≥30	95	26.3%
ASA		
1	87	24.1%
2	218	60.4%
3	55	15.2%
4	1	0.3%
Infection localization		
paramandibular	3	0.8%
submandibular	111	30.7%
perimandibular	168	46.5%
mouth floor	5	1.4%
Submental	36	10.0%
Massetericomandibular	7	1.9%
pterygomandibular	17	4.7%
parapharyngeal	14	3.9%
Infection etiology		
chronic apical parodontitis	270	74.8%
Antiresorptiv-related osteonecrosis of the jaw	7	1.9%
osteomyelitis	6	1.7%
post-osteotomy	60	16.6%
post-implantation	7	1.9%
post-osteosynthesis	2	0.6%
sialadenitis from submandibular gland	2	0.6%
Unknown	7	1.9%
Clinical symptoms		
dysphagia	330	91.4%
dyspnea	12	3.3%
stridor	4	1.1%
Clinical findings		
mandibular rim non-palpable	295	81.7%
restricted mouth opening	348	96.4%
Mallampati score		
1	29	8.0%
2	98	27.1%
3	59	16.3%
4	42	11.6%
5	1	0.3%
No ascertainable	132	36.6%
Cormack–Lehane grade		
1	167	46.3%
2	91	25.2%
3	57	15.8%
4	12	3.3%
No ascertainable	34	9.4%
Admission disposition		
surgical ward	345	95.6%
ICU	16	4.4%
surgical revision	22	6.1%
Death	1	0.3%

Abbreviations: *n* = number; % = percentage; BMI = body mass index; ASA = American Society of Anesthesiology.

**Table 2:** Correlation between infection localization and intubation modality.

Infection Localization	Intubation					
	Difficult		Regular		Total	*p* Value
	*n*/%		*n*/%			
paramandibular	0	0%	3	100%	3	** 1.000
submandibular	19	17.1%	92	82.9%	111	* 0.3703
perimandibular	35	20.8%	133	79.2%	168	* 0.6934
mouth floor	2	40.0%	3	60%	5	** 0.2610
submental	7	19.4%	29	80.6%	36	* 0.9369
massetericomandibular	3	42.6%	4	57.4%	7	** 0.1451
pterygomandibular	4	23.5%	13	76.5%	17	** 0.7554
parapharyngeal	2	14.2%	12	85.8%	14	** 0.7449
Total	72	19.9%	289	80.1%	361	

Abbreviations: *n* = number; % = percentage; significance level = 0.05. * Chi-square test. ** Fischer’s exact test.

**Table 3:** Correlation between infection-relevant clinical symptoms/findings and intubation modality.

Clinical Symptoms/Findings		Intubation					
		Difficult		Regular		Total	*p* Value
		*n*/%		*n*/%			
dysphagia	yes	3	25%	9	75%	12	** 0.7123
	no	69	19.8%	280	80.2%	349	
dyspnea	yes	3	25%	9	75%	12	** 0.7123
	no	69	19.8%	280	80.2%	349	
stridor	yes	2	50%	2	50%	4	** 0.1791
	no	70	19.6%	287	80.4%	357	
mandibular rim	not palpable	62	21.%	233	79%	295	* 0.2810
	palpable	10	15.2%	56	84.8%	66	
restricted mouth opening	yes	66	18.9%	272	81.1%	348	** 0.031
	no	6	46.1%	7	53.9%	13	
Total		72	19.9%	289	80.1%	361	

Abbreviations: *n* = number; % = percentage; significance level = 0.05. * Chi-square test. ** Fischer’s exact test.

**Table 4:** Correlation between Mallampati and Cormack–Lehane classification grade and intubation modality.

Mallampati Score	Intubation					
	Difficult		Regular		Total	*p* Value
	*n*/%		*n*/%			
1	1	3.5%	28	96.5%	29	* < 0.0001
2	10	10.2%	88	89.8%	98	
3	12	20.3%	47	79.7%	59	
4	20	47.65	22	52.4%	42	
5	1	100%	0	0%	1	
No ascertainable	28	21.2%	104	78.8%	132	
Total	72	19.9%	289	80.1%	361	
Cormack–Lehane grade	Intubation					
	difficult		regular		Total	*p* value
	*n*/%		*n*/%			
1	5	3%	162	97%	167	* < 0.0001
2	7	7.7%	84	92.3%	91	
3	27	47.4%	30	52.6%	57	
4	11	91.7%	1	8.3%	12	
No ascertainable	22	64.7%	12	35.3%	34	
Total	72		289		361	

Abbreviations: *n* = number; % = percentage; significance level = 0.05. * Cochran–Armitage trend test.

## Data Availability

The datasets generated and analyzed during the current study are not publicly available due to institutional restrictions, but are available from the corresponding author upon reasonable request.
